# Klimawandel und psychische Gesundheit. Positionspapier einer Task-Force der DGPPN

**DOI:** 10.1007/s00115-023-01457-9

**Published:** 2023-02-23

**Authors:** Andreas Heinz, Andreas Meyer-Lindenberg, DGPPN-Task-Force „Klima und Psyche“, Andreas Heinz, Andreas Meyer-Lindenberg, Mazda Adli, Barbara Bornheimer, Lasse Brandt, René Hurlemann, Sebastian Karl, Hans Knoblauch, Nina Marsh, Christoph Nikendei, Sandy Pistol, Steffi Riedel-Heller, Anna-Karina Schomburg, Kirsten Shukla, Stefan Weinmann, Franziska Welzel, Gabriel Gerlinger, Julie Holzhausen, Katja John, Isabelle Lork, Julia Sander, Annika Walinski

**Affiliations:** 1 Klinik für Psychiatrie und Psychotherapie CCM, Charité – Universitätsmedizin Berlin, 10117 Charitéplatz 1, Berlin, Deutschland; 2 Klinik für Psychiatrie und Psychotherapie, Zentralinstitut für Seelische Gesundheit, 68159 J5, Mannheim, Deutschland; 3 Deutsche Gesellschaft für Psychiatrie und Psychotherapie, Psychosomatik und Nervenheilkunde e.V., Berlin, Deutschland

**Keywords:** Empfehlungen, Psychiatrie, Nachhaltigkeit, Erderwärmung, CO_2_-Emissionen, Recommendations, Psychiatry, Sustainability, Global warming, CO_2_ emission

## Abstract

Der Klimawandel und die damit häufiger auftretenden Extremwetterereignisse wirken sich direkt negativ auf die psychische Gesundheit aus. Naturkatastrophen gehen insbesondere mit einem Anstieg von Depressionen, Angst- und Traumafolgestörungen einher. Indirekte Folgen des Klimawandels wie Nahrungsmittelknappheit, ökonomische Krisen, gewaltvolle Konflikte und unfreiwillige Migration stellen zusätzlich massive psychische Risiko- und Belastungsfaktoren dar. Klimaangst und Solastalgie, die Trauer um verlorenen Lebensraum, sind neue psychische Syndrome angesichts der existenziellen Bedrohung durch die Klimakrise. Eine nachhaltige Psychiatrie muss sich dementsprechend auf steigenden und veränderten Bedarf einstellen. Psychiatrische Behandlungsprinzipien müssen die Prävention stärker in den Blick nehmen, um das Versorgungssystem insgesamt zu entlasten. Ressourcenverschwendung und CO_2_-Ausstoß im psychiatrischen Behandlungsablauf sowie Infrastruktur müssen wahrgenommen und verhindert werden. Psychiatrische Aus‑, Fort- und Weiterbildungskonzepte sollen um die Thematik des Klimawandels erweitert werden, um Fachkräfte, Betroffene und Öffentlichkeit umfassend zu informieren, zu sensibilisieren und zu klimafreundlichem und gesundheitsförderlichem Verhalten anzuregen. Die Auswirkungen des Klimawandels auf die psychische Gesundheit müssen tiefergehend erforscht werden. Die DGPPN wird Förderer und strebt die Klimaneutralität bis 2030 an. Sie hat sich zu klimaschonenden und energiesparenden Maßnahmen im Bereich der Finanzwirtschaft, in Bezug auf den DGPPN-Kongress sowie die DGPPN-Geschäftsstelle verpflichtet.

**Zusatzmaterial online:**

Zusätzliche Informationen sind in der Onlineversion dieses Artikels (10.1007/s00115-023-01457-9) enthalten.

## Abstract (en)

Climate change and the resulting higher frequency of extreme weather events have a direct negative impact on mental health. Natural disasters are particularly associated with an increase in the prevalence of depression, anxiety and posttraumatic stress disorder. Indirect consequences of climate change, such as food shortages, economic crises, violent conflicts and forced migration, additionally represent severe psychological risk and stress factors. Climate anxiety and solastalgia, the distress induced by environmental change, are new psychological syndromes in the face of the existential threat posed by the climate crisis. Accordingly, a sustainable psychiatry must prepare for increasing and changing demands. The principles of psychiatric treatment need to focus more on prevention to reduce the overall burden on the healthcare system. Waste of resources and CO_2_ emissions in psychiatric treatment processes as well as infrastructure must be perceived and prevented. Psychiatric education, training and continuing education concepts should be expanded to include the topic of climate change in order to comprehensively inform and sensitize professionals, those affected and the public and to encourage climate-friendly and health-promoting behavior. More in-depth research is needed on the impact of climate change on mental health. The DGPPN becomes a sponsor and aims for climate neutrality by 2030 by committing to climate-friendly and energy-saving measures in the area of finance, in relation to the DGPPN congress as well as the DGPPN office.

Steigende Temperaturen und häufigere Extremwetterereignisse, das Artensterben und die zunehmende Verschmutzung von Luft, Böden und Wasser gefährden nicht nur unsere Lebensgrundlagen, sondern auch die psychische Gesundheit. Das psychiatrische Versorgungssystem muss sich auf einen steigenden Bedarf einstellen. Gleichzeitig können die in der Psychiatrie Tätigen einen Beitrag zur Bewältigung der enormen Herausforderungen leisten. Die Task-Force „Klima und Psyche“ der DGPPN hat die Evidenz zu den Auswirkungen des Klimawandels auf die Psyche zusammengefasst und Handlungsempfehlungen für eine klimaneutrale Psychiatrie erarbeitet.

## Auswirkungen des Klimawandels auf die psychische Gesundheit

Es ist inzwischen gut belegt, dass sich der Klimawandel direkt und indirekt negativ auf die psychische Gesundheit auswirkt [1]. Die Zunahme von Extremwetterereignissen wie Hitzewellen oder Überschwemmungen kann zur Verschlechterung bestehender oder zum Ausbruch neuer psychischer Erkrankungen wie PTBS, Angststörungen oder Depressionen führen: Eine Metaanalyse zeigt, dass Menschen, die Naturkatastrophen miterlebt haben, ein fast doppelt so hohes Risiko für eine psychische Erkrankung aufwiesen, verglichen mit Menschen ohne eine solche Erfahrung [2]. Durch die menschengemachte Zerstörung von Lebensräumen und -grundlagen entstehen darüber hinaus begründete Zukunftsängste [3], ökonomische Krisen, Nahrungsmittelunsicherheit, gewaltvolle Konflikte und Vertreibung von Menschen, die massive Belastungs- und Risikofaktoren für die psychische Gesundheit darstellen [4].

### Direkte Auswirkungen auf die Psyche

Der Klimawandel wirkt sich über zunehmende Hitze und Naturkatastrophen wie Überschwemmungen, Dürren, Stürme und Brände direkt auf die psychische Gesundheit aus. Auch die Luftverschmutzung hat einen direkten, negativen Effekt auf die Psyche.

#### Luftverschmutzung

Der Klimawandel geht Hand in Hand mit der Industrialisierung, Urbanisierung und Luftverschmutzung. Luftverschmutzung wirkt schädlich auf die kognitiven Funktionen und kann Aufmerksamkeit, visuokonstruktive Fähigkeiten, Gedächtnis, Rechenleistung, Leseverständnis sowie verbale und nonverbale Intelligenz beeinträchtigen [5–7]. Eine wachsende Zahl an Studienbefunden weist außerdem auf einen Zusammenhang zwischen Luftverschmutzung und Risiko für psychische Erkrankungen wie z. B. Depression, Aufmerksamkeitsdefizit-/Hyperaktivitätsstörung (ADHS) und Schizophrenie hin [8–10]. In einer großen Metaanalyse konnte ermittelt werden, dass ein Anstieg der Feinstaubbelastung kurzfristig mit mehr psychiatrischen Notfällen und erhöhter Suizidalität in den folgenden Tagen einhergeht. Über einen längeren Zeitraum zeigt sich außerdem eine steigende Depressionsprävalenz im Zusammenhang mit erhöhter Feinstaubbelastung [11].

#### Hitze

Der Klimawandel führt weltweit zum Anstieg der Durchschnittstemperaturen, auch die Häufigkeit und Schwere von Hitzewellen nimmt zu [12]. Psychische Erkrankungen gehören zu den wichtigsten Risikofaktoren für hitzebedingte Todesfälle. Sie verdreifachen das Mortalitätsrisiko während Hitzewellen und sind damit schwerwiegender als kardiovaskuläre oder Lungenerkrankungen [13]. Das höchste hitzebedingte Mortalitätsrisiko haben Menschen mit substanzbezogenen Süchten und organischen psychischen Störungen wie z. B. Demenzen [14]. Diese besonders vulnerablen Patienten können sich häufig nicht selbstständig und effektiv vor Hitze schützen. Auch die Zahl der Suizide steigt mit den Temperaturen an [15].

Eine 2021 veröffentlichte Metaanalyse zeigt, dass pro 1‑Grad-Celsius Temperaturanstieg ein 0,9 % höheres Risiko für psychische Erkrankungen existiert [14]. Die psychische Belastung durch Hitze drückt sich außerdem in einem deutlichen Anstieg von (Not‑)Aufnahmen in psychiatrische Kliniken aus [15].

Forscher vermuten einen kausalen Zusammenhang zwischen Hitze und Aggressivität [16]. Dazu passt, dass psychiatrische Kliniken mehr aggressive Zwischenfälle verzeichnen, je höher die Temperaturen sind [17].

#### Extremwetter und Naturkatastrophen

Überschwemmungen, Brände, Stürme und Dürren sind Extremwetterereignisse, die aufgrund des Klimawandels zukünftig immer häufiger und stärker auftreten. Sie können zu Naturkatastrophen werden, die eine Bedrohung der körperlichen Unversehrtheit sowie die Zerstörung von Existenzgrundlagen und kritischer Infrastruktur zur Folge haben. Entsprechend können sie auch massive psychische Belastungen für die Betroffenen darstellen. Eine mangelnde oder unterbrochene Gesundheitsversorgung stellt einen weiteren Risikofaktor für das Entstehen psychischer Störungen und die Verschlechterung bestehender psychischer Erkrankungen nach Naturkatastrophen dar. Auch ein notgedrungener Wohnortwechsel und der Verlust von Eigentum, Arbeitsplatz und sozialer Unterstützung gehören zu den länger andauernden psychosozialen Stressoren, die die psychische Gesundheit der Menschen in betroffenen Regionen gefährden [1].

Die Intensität einer Naturkatastrophe bzw. der Grad der Betroffenheit einer Person steigert dabei die Schwere der psychischen Symptomatik, die noch Jahre über die akute Notlage hinaus bestehen kann [18–20]. Insbesondere die Prävalenz posttraumatischer Belastungsstörungen (PTBS) steigt nach zerstörerischem Extremwetter massiv an [21, 22]. Beispielsweise litten unter Betroffenen einer Flutkatastrophe in England ein Jahr nach dem Ereignis 36,2 % der Bevölkerung in der Region unter PTBS [23]. Auch wies fast jeder dritte Bewohner von New Orleans nach Hurricane Katrina im Südosten der USA 2005 Symptome einer PTBS auf [24].

Generalisierte Ängste, Depressionen und erhöhte Suizidraten sind ebenso Folge von verschiedenen Extremwettern und Naturkatastrophen. Verschiedene Studien zeigen eine deutlich erhöhte Prävalenz affektiver Störungen nach Busch- und Waldbränden [22, 25, 26]. Etwa die Hälfte der Bewohner in New Orleans litt in den 30 Tagen nach Hurricane Katrina unter einer affektiven Störung. Viele berichten außerdem von Suizidgedanken [24, 27]. Noch ein Jahr nach großen Überschwemmungen leidet etwa ein Viertel der Betroffenen an Angsterkrankungen und ein Fünftel unter Depressionen [23]. Dasselbe Muster zeigt die australische Forschung in Verbindung mit der chronischen Dürre des Landes [1, 20, 28–30].

Einzelne Studien fanden außerdem Belege für vermehrten Alkohol- und Substanzgebrauch und -missbrauch sowie gehäufte häusliche Gewalt infolge von Naturkatastrophen [1].

#### Angst vor der Zukunft

Der Begriff „eco distress“ wird verwendet, um eine Reihe emotionaler Reaktionen angesichts der Umweltzerstörung der Erde zu beschreiben. Diese negativen Emotionen in Bezug auf den Klimawandel und den Verlust an Biodiversität betreffen nicht nur psychisch Erkrankte oder direkt von Naturkatastrophen betroffene Menschen [1, 3]. Gemeint sind Hoffnungslosigkeit, Traurigkeit, Schuldgefühle, Wut, Sorgen, Angst und Panik der allgemeinen Bevölkerung. Die Verhaltensweisen reichen von Verleugnung und Verdrängung, Starre aufgrund von Überforderung und Hilflosigkeit bis hin zu aufopferndem Aktivismus. Im Kontext des Bewusstwerdens des Klimawandels ist neben „eco distress“ eine Reihe neuer Begriffe entstanden, die die psychologischen und emotionalen Reaktionen beschreibt. Das Phänomen der Klimaangst „climate anxiety“ beschreibt die Erwartung, in Zukunft selbst direkt vom Klimawandel betroffen zu sein, wobei die Ungewissheit in Bezug auf die Art, den Zeitpunkt und den Ort zusätzlich belastet [31]. „Solastalgie“ bezeichnet die mit der Zerstörung der eigenen Heimat bzw. Umwelt einhergehende Trauer angesichts des Verlusts von Orten, Aktivitäten oder Traditionen aufgrund des Klimawandels [32].

### Indirekte Folgen des Klimawandels auf die Psyche

Langzeitdürren, Überschwemmungen, Brände und Wirbelstürme, deren Häufigkeit und Dauer im Zuge des Klimawandels voraussichtlich zunehmen werden, sind assoziiert mit der Verschlechterung wirtschaftlicher Bedingungen, können den Zugang zu Nahrung und Trinkwasser für große Bevölkerungsgruppen erschweren und zum Teil zur Migration zwingen [20]. Diese indirekten Effekte des Klimawandels werden vorrausichtlich auch zu Verteilungskonflikten über natürliche Ressourcen führen und zusätzlich starke psychische Belastungen hervorrufen. Darüber hinaus ist der Klimawandel auch Gegenstand begründeter Sorgen und Zukunftsängste („eco distress“) vieler Menschen weltweit, die (noch) nicht unmittelbar betroffen sind.

#### Nahrungsmittelunsicherheit

Durch Dürren oder die Zerstörung oder Veränderung landwirtschaftlicher Nutzflächen kann Nahrungsknappheit und eine Verringerung des Nährstoffgehalts und somit Qualität von Getreidearten entstehen, was Mangelerscheinungen zur Folge haben kann [20]. Metaanalysen zeigen, dass Mangelernährung sich in psychischen Symptomen wie Fatigue, Gedächtnisschwäche oder depressiven Verstimmungen äußern kann. Eine gesunde Ernährung hingegen unterstützt psychische Gesundheit, kognitive Leistung, Stimmung und Stressempfindlichkeit. Besonders vulnerabel für die Folgen von Mangelernährung auf die psychische Gesundheit sind Frauen, ältere Menschen und Kinder, bei denen ein erhöhtes Risiko für die Entwicklung von Depressionen oder ADHS entstehen kann [33].

#### Flucht und Migration

Die Zerstörung von Lebensräumen, Nahrungsmittel- und Trinkwasserknappheit und nicht zuletzt auch ökonomische und institutionelle Krisen können Staaten destabilisieren, zu Konflikten und Kriegen und dazu führen, dass Menschen klimawandelbedingt migrieren müssen. Die Flucht an sich, die daraus folgende Unterbrechung sozialer Netzwerke und Arbeitsplatzunsicherheit stellen erhebliche Belastungsfaktoren dar, die die psychische Gesundheit gefährden und das Risiko für Angststörungen und affektive Erkrankungen erhöhen [34, 35]. Studien in Bangladesch zeigten, dass mit umweltbedingter Umsiedlung oft auch eine Verschlechterung der Wohn- und Arbeitsbedingungen einhergeht und Menschen neben ökonomischen auch nichtökonomische Verluste wie Identität und Zugehörigkeitsgefühl erleben [36]. Hinzu kommt, dass Geflüchtete ein höheres Risiko für psychotische Erkrankungen haben [37]. Negative Erfahrungen im Anpassungsprozess nach der Migration begünstigen zudem depressive Symptome und erhöhen das Suizidrisiko [38].

#### Klimaungerechtigkeit

Klimaungerechtigkeit liegt darin, dass diejenigen Menschen, die am wenigsten zur Verursachung der Klimakrise beitragen, oft am schwersten betroffen sind. Die psychischen Auswirkungen des Klimawandels sind dahingehend ungleich verteilt, dass bestimmte Individuen oder Bevölkerungsgruppen besonders von den Umweltveränderungen betroffen sind – aufgrund geografisch bedingt erhöhter Exposition, des zugrunde liegenden Gesundheitszustands oder begrenzter Anpassungs- oder Bewältigungskapazitäten [39]. Auch soziale und wirtschaftliche Faktoren können das Ausmaß der psychischen Folgen beeinflussen und bestimmte Menschen benachteiligen. Für indigene Bevölkerungen, Geflüchtete und Migranten, ethnische Minderheiten, Obdachlose und vulnerable Populationen in ärmeren Ländern hat der Klimawandel besonders starke bis hin zu existenzielle Auswirkungen; eine besondere Neigung, neue psychische Erkrankungen oder eine Verschlechterung bestehender psychischer Probleme zu entwickeln, zeigt sich in vielen Studien für Frauen und Kinder, Menschen mit niedrigem sozioökonomischem Status, psychisch Erkrankte und Menschen mit weniger sozialen Netzwerken [40–42]. Weiter sind insbesondere Arbeiter und Landwirte durch Wetter- und Umweltveränderungen oftmals in ihrer wirtschaftlichen Existenz bedroht, was zu starker Verzweiflung und erhöhten Suizidraten führen kann [1, 29]. Hinzu kommt, dass die psychische Gesundheit von Menschen, die in Städten oder ärmeren Stadtteilen leben, durch weniger verfügbare Grünflächen, mehr Hitze und Luftverschmutzung häufig besonders gefährdet ist [43, 44] – ein Aspekt, der angesichts der zunehmenden Urbanisierung zusätzlich an Relevanz gewinnt. Insgesamt verschärfen sich durch den Klimawandel weltweit bestehende soziale, wirtschaftliche und gesundheitliche Ungleichheiten [45]. Die Kinder und Jugendlichen von heute werden die größte Last der gesundheitlichen Auswirkungen tragen und sind daher in besonderem Maße von einer intergenerationalen Ungerechtigkeit betroffen [46].

## Handlungsempfehlungen für eine klimaneutrale Psychiatrie

Angesichts häufigerer psychischer Erkrankungen und neuer Belastungen durch den Klimawandel ist mit einer Zunahme des psychiatrischen Versorgungsbedarfs zu rechnen. Insbesondere im Bereich der Traumafolgestörungen, Angsterkrankungen und Depressionen wird der Behandlungsbedarf weiter steigen. Die zu erwartende klimabedingte Zunahme von Migration erfordert zudem kultursensible Angebote. Die Psychiatrie muss sich auf Versorgungskonzepte einstellen, die nachhaltig sind und dem steigenden und sich verändernden Bedarf gerecht werden.

Die Task-Force „Klima und Psyche“ hat sich ausführlich mit den Handlungsmöglichkeiten im Umgang mit der Klimakrise innerhalb der Psychiatrie auseinandergesetzt. Die Task-Force hat für die Bereiche „Versorgung“, „Forschung“ und „Aus‑, Fort- und Weiterbildung“ Handlungsebenen definiert und Empfehlungen ausgearbeitet, die zur Nachhaltigkeit der Psychiatrie beitragen können. Im Folgenden werden die Handlungsempfehlungen für die genannten Bereiche zusammengefasst.

Eine Zusammenfassung mit *zehn zentralen Handlungsempfehlungen* für in der Psychiatrie Tätige, über verschiedene Handlungsebenen hinweg, ist im *Anhang 1 im Online-Supplement* dargestellt.

### Versorgung

Im Sinne einer nachhaltigen Psychiatrie soll es Ziel sein:

- die Emission von Treibhausgasen und Materialverbrauch in den klinischen Einrichtungen zu reduzieren,
- die psychiatrisch-psychotherapeutische Behandlungskette und das Hilfesystem effektiv und ressourcenschonend zu gestalten,
- Behandlungsangebote an Veränderungen des psychiatrischen Handlungsbedarfs bzw. des Diagnosespektrums anzupassen.

#### Infrastruktur, Material und Abläufe anpassen

Psychiatrische Kliniken und Praxen stoßen als Teil des Gesundheitssektors einen relevanten Anteil der insgesamt im deutschen Gesundheitssektor anfallenden ca. 0,71 t CO_2_ pro Kopf aus [47–49]. Der stationäre Bereich ist wesentlich energieintensiver als der ambulante. In psychiatrischen Fachkliniken wie auch in psychiatrischen Abteilungen in Allgemeinkrankenhäusern können dieselben auf Energiemanagement, Mobilität, Recycling, Abfall, Ressourcenverbrauch, Lebensmittel, Beschaffung und Gebäude zielenden Maßnahmen wie in Kliniken allgemein und anderen Abteilungen ergriffen werden. Diese Maßnahmen sind beschrieben im Leitfaden „Klimaschutz in Kliniken verankern“ des Projektes „KLIK – Klimamanager für Kliniken“ [50] und im Rahmenwerk „Klimagerechte Gesundheitseinrichtungen“ der Allianz KLUG (Klimawandel und Gesundheit; [47]). Für vertragsärztliche Praxen sind im letztgenannten Dokument spezifische Anregungen enthalten. Die Infrastruktur von Kliniken und Praxen muss nachhaltig gestaltet, zur Energiewende genutzt und darüber hinaus an erwartbare Umweltveränderungen wie höhere Außentemperaturen und Hitzewellen angepasst werden. Die Bereiche Mobilität und Lebensmittelversorgung sollten auf nachhaltige Alternativen umgestellt (Förderung der ÖPNV-Nutzung, vermehrte biologische und vegetarische Lebensmittel) und der Verbrauch jeglicher Ressourcen und Materialen im Gebäudemanagement und Behandlungsablauf reduziert werden. Zur Vermeidung von Ressourcenverschwendung im Behandlungsablauf dienen u. a.:

- leitliniengerechte Optimierung des Medikamenten- und Materialverbrauchs (z. B. Abdosierung prüfen [51]),
- Minimierung des Einsatzes von Einwegprodukten,
- Möglichkeiten der Digitalisierung zur Behandlung nutzen,
- Reduktion wenig effektiver Prozesse,
- Ambulantisierung der Behandlung.

Auch organisatorische Veränderungen wie die Einführung einer klimabeauftragten Person, regelmäßige Verbrauchsanalysen und die Berücksichtigung von Nachhaltigkeitskriterien bei der Beschaffung und Finanzierung spielen eine wesentliche Rolle beim Klimaschutz durch Kliniken und Praxen. Marketing für Nachhaltigkeit über Klinik- oder Praxiskommunikation (z. B. Klimasprechstunde) und Transparenz (z. B. über den Energieverbrauch) sowie Schulungen tragen zur Sensibilisierung und Verhaltensänderung bei.

Eine ausführliche Auflistung empfohlener Maßnahmen auf verschiedenen Ebenen der *Infrastruktur und Abläufe *von Kliniken und Praxen sowie Forschungseinrichtungen ist im *Anhang 2 im Online-Supplement *zu finden.

#### Behandlungskette optimieren

Um den zu erwartenden Anstieg des Bedarfs an psychiatrischer Versorgung zu bewältigen, muss das Versorgungssystem effizient gestaltet sein und früh intervenieren. Je weniger Bedarf an psychiatrisch-psychotherapeutischer Behandlung auftritt und je zielgenauer dieser Bedarf gedeckt wird, desto weniger Ressourcenverbrauch findet statt. Daher sind weitgehende Strategien erforderlich, um die psychiatrisch-psychotherapeutische Krankheitslast zu senken, was den Fokus auf Prävention und die Deckung wesentlicher Lebensbedürfnisse, insbesondere bei Menschen mit psychischer Vulnerabilität, legt.

Um gesundheits- und klimabewusstes Verhalten anzuregen und damit zu einer Verringerung der Folgen des Klimawandels für die Gesundheit beizutragen, ist es wichtig, individuelles und soziales Wohlbefinden bzw. soziales Kapital zu fördern [52]. Weiter ist es essenziell, Betroffenen und Tätigen im Versorgungssystem dazu zu verhelfen, die eigene psychische Gesundheit zu fördern bzw. zu entwickeln. Es ist davon auszugehen, dass psychiatrische Hilfen, denen ein Empowerment aller Beteiligten gelingt, resilienter sind, effizienter arbeiten und dadurch auch weniger CO_2_-intensiv sind. Wenn zudem soziale Determinanten psychischer Gesundheit stärker berücksichtigt werden, sind die Ergebnisse nachhaltiger und es tritt weniger Bedarf an stationärer und ressourcenintensiver Behandlung auf.

Zur Verringerung von Morbidität, und somit dem Behandlungsbedarf, können beitragen:

- Empowerment und Ownership, d. h. Förderung von Gesundheitskompetenz, Selbstsorge, Peer-Support, Zugang zu Psychotherapie,
- Förderung sozialer Netzwerke und unterstützender sozialer Beziehungen,
- Verringerung von Obdachlosigkeit und sozialer Isolation,
- Förderung von Beschäftigung bei Menschen mit psychischer Erkrankung,
- Planung ausreichender Grünflächen in psychiatrischen Einrichtungen.

Weiterhin muss die Widerstandsfähigkeit des psychosozialen Versorgungssystems gestärkt werden, damit es auf Unterbrechungen aufgrund klimabedingter Katastrophen vorbereitet ist und die Versorgung im Katastrophenfall fortgesetzt werden kann, z. B. durch die Bereitstellung digitaler psychosozialer Dienste.

Detaillierte Empfehlungen zur *Optimierung der Behandlungskette* für eine nachhaltigere psychiatrisch-psychotherapeutische Versorgung sind im *Anhang 3 im Online-Supplement *zu finden.

#### Neue Behandlungsangebote schaffen

Eine klimaneutrale psychiatrische Versorgung muss die klimawandelbedingten Veränderungen des Behandlungsbedarfs vorausdenken. So ist eine Anpassung des Angebots dahingehend notwendig, dass das häufigere Auftreten psychischer Krisen und vermehrte Angsterkrankungen (auch: Klimaängste), Traumafolgestörungen und das Vorkommen sozialer Isolation in Folge von Katastrophenereignissen versorgt und eine Chronifizierung verhindert werden können. Hierfür kommt auch der Aufbau von Spezialambulanzen für Krisen mit Bezug zum Klimawandel infrage.

Angesichts der möglichen Zunahme klimabedingter Migration müssen darüber hinaus kultursensible Angebote geschaffen und Sprachmittlung in der Behandlung gewährleistet werden, um die Unter- oder Fehlversorgung von Personen mit Migrationshintergrund oder geflüchteten Personen zu vermeiden. Eine bessere Kooperation mit somatischen medizinischen Fächern wird notwendig sein, um die Morbidität somatischer Erkrankungen und das Risiko von Folgeerkrankungen bei der vulnerablen Gruppe psychisch Erkrankter, insbesondere schwer psychisch Erkrankter, bei älteren Menschen und Kindern zu verringern.

Weitere Details zu möglichen *neuen Behandlungsangeboten* für eine nachhaltigere psychiatrisch-psychotherapeutische Versorgung sind im *Anhang 4 im Online-Supplement *aufgeführt.

### Forschung und Wissenschaft

Die psychiatrische Forschung muss durch die Entwicklung von Präventions- und Interventionsmaßnahmen einen Beitrag im Sinne einer nachhaltigen Psychiatrie leisten. Dringender Forschungsbedarf besteht zu den neu auftretenden Syndromen wie „Solastalgie“ und „Klimaangst“. Die Auswirkungen des Klimawandels speziell auf die psychische Gesundheit von Kindern und Jugendlichen sind ebenfalls noch unzureichend untersucht.

Auch Forschungseinrichtungen selbst arbeiten jedoch ressourcenintensiv und können an vielen Stellen klimaneutraler werden. Für den Bereich Forschung und Wissenschaft existieren bereits diverse Leitfäden universitärer und außeruniversitärer Forschungseinrichtungen, die sich dem Thema „Nachhaltigkeitsmanagement“ widmen. Beispiele sind die Handreichung des vom Bundesministerium für Bildung und Forschung (BMBF) geförderten LeNa-Projekts [53], die Checklisten und Handlungsempfehlungen der Deutschen Gesellschaft für Nachhaltigkeit an Hochschulen (DG HochN; [54, 55]) oder die Handlungsempfehlungen von Fraunhofer Klimaneutral 2030 [56]. Die bestehenden Empfehlungen zu nachhaltiger Forschung lassen sich zunächst in die vier Dimensionen „Institutionelle Verankerung und Governance“, „Forschungsförderung“, „Forschungsprozess“ und „Vernetzung“ einsortieren.

Auf der Ebene des Forschungsprozesses sind neben der Reduktion unnötiger Ressourcenverschwendung insbesondere auch die langfristigen und ggf. ungünstigen Wirkungen und Konsequenzen zu bedenken, die sich aus dem Forschungsprozess und den Ergebnissen ergeben können [53, 57].

Die Maßnahmen, die auf Ebene von *Infrastruktur*, *Abläufen und materieller Veränderungen von Forschungseinrichtungen* empfohlen werden können, sind zu großen Teilen deckungsgleich mit denen für psychiatrische Kliniken und Praxen im *Anhang 2 im Online-Supplement*.

Eine ausführliche Auflistung empfohlener *Strategien für eine nachhaltige Forschung* ist im *Anhang 5 im Online-Supplement* zu finden. *Literaturverweise* auf bereits *bestehende Standards und Richtlinien* für eine nachhaltige Forschung finden sich im *Anhang 6 im Online-Supplement*.

### Aus‑, Fort- und Weiterbildung

Informationen zum Klimawandel und zu seinen gesundheitlichen Konsequenzen sollten Teil der regulären Ausbildung von Ärztinnen, Ärzten und weiteren medizinischen Fachkräften werden. Als Reaktion auf einen Beschluss der 93. Gesundheitsministerkonferenz (GMK) im Jahr 2020 hat die Bundesärztekammer (BÄK) die „Auswirkungen des Klimawandels auf die Gesundheit“ in die Musterweiterbildungsordnung (MWBO) für Ärztinnen und Ärzte aufgenommen [58]. Die inhaltliche Ausgestaltung blieb bisher jedoch noch unbestimmt.

Grundlagenwissen zur Entstehung des menschengemachten Klimawandels sowie Details zum Einfluss auf die menschliche Gesundheit, insbesondere der psychiatrischen Aspekte, sollten obligatorischer Inhalt der Aus‑, Fort- und Weiterbildung werden. Ebenso sollten die Folgen für vulnerable Gruppen und die Klimaungerechtigkeit adressiert werden. Die Rolle und Verantwortung des Gesundheitswesens müssen deutlich gemacht werden.

Die genannten Lerninhalte sollen in die Kurrikula von Ärztinnen und Ärzten in fachärztlicher Weiterbildung für Psychiatrie und Psychotherapie, Kinder- und Jugendpsychiatrie und -psychotherapie, psychosomatische Medizin sowie von Psychologischen Psychotherapeutinnen und -therapeuten in Ausbildung eingefügt werden. Auch Auszubildenden in Gesundheits- und Krankenpflege, Patientinnen und Patienten sowie der Öffentlichkeit sollten entsprechende Informationsveranstaltungen zugänglich gemacht werden.

Für die Durchführung von Lehrveranstaltungen gilt es, grundsätzlich zu prüfen, ob diese als Onlineveranstaltung angeboten werden können. Eine detaillierte Auflistung von, in Zukunft notwendigen, *Inhalten für die Aus‑, Fort- und Weiterbildung *findet sich im *Anhang 7 im Online-Supplement*.

Wichtige *Materialien und Ressourcen* als Grundlage der inhaltlichen Ausgestaltung werden im *Anhang 8 im Online-Supplement *dargestellt.

Im *Anhang 9 im Online-Supplement *sind Maßnahmen für eine klimabewusstere Durchführung von *Präsenzveranstaltungen* gelistet.

## Aktionsplan der DGPPN für eine nachhaltige Fachgesellschaft

Die DGPPN bekennt sich im Einklang mit dem Deutschen Ärztetag zur Pflicht, die „Auswirkungen des Klimawandels klar zu benennen, die gesundheitliche Bedrohung durch den Klimawandel aufzuzeigen, Gegenmaßnahmen einzufordern und mit dazu beizutragen, dass sich das Gesundheitssystem auf die Bewältigung der Folgen des Klimawandels vorbereitet und bei jeglichem Handeln zum Wohle der Gesundheit klimaschädliche Auswirkungen vermeidet“ [58].

Mit über 10.000 Mitgliedern ist die DGPPN die größte medizinisch-wissenschaftliche Fachgesellschaft für Fragen der psychischen Erkrankungen in Deutschland. Regelmäßige Gremienarbeit leisten die 35 Fachreferate, der 19-köpfige Vorstand, die 11 Beiratsmitglieder sowie das Trialogische Forum. Hinzu kommt der jährliche DGPPN-Kongress mit seinen mehreren Tausend Teilnehmenden sowie weitere Veranstaltungs‑, Fort- und Weiterbildungsformate, die von der DGPPN durchgeführt werden. Das gesamte Spektrum an Aktivitäten wird unterstützt durch eine Geschäftsstelle mit mehr als 20 Mitarbeitenden.

Der Aktionsplan der DGPPN für eine nachhaltige Fachgesellschaft umfasst die Bereiche „Bewusstseinsbildung“, „Interessenvertretung“, „Forschungsförderung“, „DGPPN-Kongress“, „Finanzen“ und die DGPPN-Geschäftsstelle.

Dazu gehört, dass die Fachgesellschaft sich verpflichtet, den Zusammenhang von Klimawandel und psychischen Erkrankungen in verschiedenen Formaten und Veranstaltungen darzustellen und darüber zu informieren. Der DGPPN-Kongress dient hierzu als Plattform und soll ab 2030 möglichst klimaneutral abgehalten werden – d. h. CO_2_-Ausstoß zu vermeiden, zu reduzieren und zu kompensieren. Zusätzlich soll geprüft werden, ob das Vereinsvermögen, Geldflüsse und Versicherungen der DGPPN zu nachhaltigen und nach ethischen Kriterien arbeitenden Anbietern umgezogen werden können. Weiterhin möchte die Fachgesellschaft das Forschungsfeld „Klima und psychische Erkrankungen“ weiter vorantreiben, um Antworten auf die aktuellen und zukünftigen Herausforderungen in der Gesundheitsversorgung zu finden.

Diese und weitere *Maßnahmen der DGPPN* werden im *Anhang 10 im Online-Supplement *ausführlich beschrieben.

## Supplementary Information




