# Supplementary material for: Climate change and mental health. Position paper of a task force of the DGPPN
Source: Nervenarzt. 2023 Feb 23;94(3):225–33. [Article in German] doi: 10.1007/s00115-023-01457-9 (PMC9992044; doi:10.1007/s00115-023-01457-9)
Supplement: Supplementary file 1 [file 115_2023_1457_MOESM1_ESM.pdf]

Online Supplement

Inhalt

A1	Zehn zentrale Handlungsempfehlungen für eine klimaneutrale Psychiatrie	2
A2	Infrastruktur, Abläufe und materielle Veränderungen (Kliniken, Praxen und Forschungseinrichtungen)	3
A3	Psychiatrisch-psychotherapeutische Behandlungskette optimieren	8
A4	Neue Behandlungsangebote schaffen – Anpassung an mögliche Veränderungen des psychiatrischen Handlungsbedarfs und Diagnosespektrums im Rahmen des Klimawandels	9
A5	Strategien einer nachhaltigen Forschung	10
A6	Standards und Richtlinien für nachhaltige Forschung	12
A7	Inhalte einer Aus-, Fort- und Weiterbildung zu „Klimawandel und Psychiatrie/Psychotherapie“	13
A8	Materialien, Ressourcen und Quellen für Aus-, Fort- und Weiterbildung	15
A9	Checkliste zur Organisation einer klimabewussten Aus-, Fort- und Weiterbildung	17
A10	Aktionsplan für eine nachhaltige Fachgesellschaft	19
	Literatur	25

A1 Zehn zentrale Handlungsempfehlungen für eine klimaneutrale Psychiatrie

1. ***Mental Health in all Policies:*** Mehr Prävention sowohl in der Psychiatrie wie auch sektorübergreifend, z.B. durch Planung ausreichender und zugänglicher Grünflächen in Einrichtungen des psychiatrischen Hilfesystems, wie Zugang zu verschatteten Gärten mit Bäumen statt nur „Freiflächen" bei Unterbringung nach PsychKG, Verringerung von Obdachlosigkeit und sozialer Isolation, Förderung von Beschäftigung bei Menschen mit psychischer Erkrankung.

2. Mehr ***Empowerment*** innerhalb des psychiatrischen Versorgungssystems (z.B. Förderung von Gesundheitskompetenz, Selbstsorge, *Peer Support* und Zugang zu Psychotherapie).

3. Integration der Thematik „Klima und Psyche" in psychiatrischer **Aus-/Fort-/Weiterbildung**, Behandlung und Gestaltung der Hilfesysteme sowie Forschung (Erforschung der Folgen des Klimawandels und Entwicklung von Präventions- und Interventionsmaßnahmen).

4. **Klinik- und Praxisinfrastruktur zur Energiewende** nutzen (z.B. Installation von Photovoltaik, finanzierbar z.B. durch *Contracting*), Bezug von Ökostrom, Dämmung und Verschattung statt Klimaanlage (wo möglich), energetische Sanierung unter Beachtung von „grauer Energie", d.h. Energie für Herstellung, Transport, Lagerung, Verkauf und Entsorgung).

5. **Adaptation** der Institutionen in der Psychiatrie an erwartbare Umweltveränderungen, z.B. Anpassung der Klinik- und Praxisinfrastruktur an Hitze, Anpassung an Zunahme von Depressionen, Angsterkrankungen und psychotischen Erkrankungen.

6. **Vermeidung von Ressourcenverschwendung im Behandlungsablauf:** Leitliniengerechte Optimierung des Medikamenten -und Materialverbrauchs (z. B. Abdosierung prüfen, Einsatz von Einwegprodukten minimieren, Möglichkeiten der Digitalisierung nutzen), Reduktion wenig gewinnbringender Prozesse.

7. **Reduktion von motorisiertem Individualverkehr,** z.B. Mobilität durch Möglichkeiten der Digitalisierung nutzen (z.B. durch digitale Behandlungsoptionen, digitale Besprechungen/ Konferenzen), um zur Reduktion der CO_2-Emissionen und Schadstoffbelastung beizutragen.

8. Umsetzung einer vornehmlich **pflanzenbasierten Verpflegung** mit einem maximal geringen Anteil tierischer Produkte in Kliniken (Orientierung an *Planetary Health Diet* oder Empfehlungen der Deutschen Gesellschaft für Ernährung e.V.), da die pflanzenbasierte Ernährung dem Umweltschutz und der allgemeinen Gesundheit dienlich ist.

9. Berücksichtigung von **Nachhaltigkeitskriterien bei der Beschaffung und Finanzierung** (z.B. bei Anlageentscheidungen, im Einkauf oder bei der Forschungsfinanzierung).

10. **Marketing für Nachhaltigkeit über** Klinik- oder Praxiskommunikation (z.B. Klimasprechstunde).

A2 Infrastruktur, Abläufe und materielle Veränderungen (Kliniken, Praxen und Forschungseinrichtungen)[2]

Bereiche	Was	Nutzen
**Energie**	Prinzip *Green IT*:<br>– Nutzung erneuerbarer Energien: Umstellen auf Ökostrom und Photovoltaik<br>– Bei Anschaffungen auf Nachhaltigkeit achten<br>– Ressourcensparende Softwareprogrammierung nutzen	Nachhaltiger
	Energiesparlampen oder LEDs nutzen	Sind langlebiger, sparen Energie und Müll
	Stoßlüften und Thermostat ausschalten (bei gekippten Fenstern geschieht wenig Luftaustausch, Wände kühlen aus)	Reduziert Energieverbrauch, verbessert Luftqualität
	Nicht benutzte Geräte ausschalten (automatisches Ausschalten von Computern und anderen elektronischen Geräten über Nacht)	Reduziert Verbrauch
	Bewegungsmelder nutzen, wo möglich	Spart Strom und ist hygienischer
	Gemeinschaftsnutzung von Technik/Geräten/Räumen, um Leerlauf zu vermeiden	Spart Ressourcen, reduziert Verbrauch
	Lüftung:<br>– Reduzierung der Lüfterleistung<br>– Laufzeitoptimierung und Abschaltung<br>– Einsatz von Präsenzmeldern	Reduziert Verbrauch
	Kühlung:<br>– Anhebung der Kühlwassertemperatur<br>– Abgleich der Volumenströme<br>– Kälteerzeugung durch Freie Kühlung<br>– Temperaturerhöhung in Serverräumen<br>– Bedarfsoptimierung<br>– Einbau automatischer Türschließer	Reduziert Verbrauch
	Heizung:<br>– Bedarfsoptimierung<br>– Hydraulischer Abgleich<br>– Kesselmanagement<br>– Effizientere Pumpen<br>– Optimierung der Infrastruktur<br>– Warmwasserspeicherung im Leitungsnetz	Reduziert Verbrauch
	Beleuchtung:<br>– Präsenzmelder<br>– Flurlampen auf LED umstellen<br>– Beleuchtung Verteilerschränke<br>– Dimmbare LED-Beleuchtung<br>– Außenbeleuchtung mit LED	Reduziert Verbrauch
	Contracting	Umsetzung von Energiesparmaßnahmen

2 Die Handlungsempfehlungen für die klinische Versorgung (Anhang 2–4) erheben keinen Anspruch auf Vollständigkeit. Maßnahmen, die nachhaltig, vernünftig und evidenzbasiert sind, sind gleichzeitig auch ressourcen- und klimaschonend und können die Liste an Handlungsmöglichkeiten sinnvoll ergänzen.

Bereiche	Was	Nutzen
**Mobilität**	ÖPNV-Tickets für Mitarbeitende finanziell unterstützen	Nachhaltigkeitsanreiz
	Dienstreisen:<br>– Anzahl reduzieren, wenn möglich<br>– Nachhaltige Mobilität nutzen: Fahrrad, ÖPNV statt Auto, Bahn statt Flugzeug<br>– CO_2-Kompensation bei Flügen	Spart CO_2
	Telefon- und Videokonferenzen statt *Face-to-Face-Meetings*	Spart Reisekosten, Ressourcen, Zeit und Geld
	Job-Bikes/E-Mobilität fördern	Nachhaltiger
	Arbeitswege: Möglichst „grüne Wege" per Rad, Fuß o. ÖPNV (durch Gärten/Parkanlagen, bepflanzte Straßen) nutzen, um zum Ziel zu kommen: statt Schnelligkeit auf Entschleunigung zielen	Gesundheitsförderlich, nachhaltiger
**Abfall und Recycling**	Mülltrennung: Müll nach Plastik, Papier und Restmüll trennen und Mülltonnen hierzu kennzeichnen	Nachhaltiger
	Umweltgerechte Entsorgung von Geräten/Technik und Schadstoffen	Nachhaltiger
	Abfall vermeiden:<br>– Reduzieren von *wasteful/low value activities*<br>– Mehrweg- statt Einwegprodukte nutzen<br>– Kugelschreiberminen austauschen, statt Stift wegwerfen<br>– Rückgabe/Rücknahme von Verpackungsmaterial<br>– Umgang mit Werbesendungen optimieren, z.B. Eintrag in https://www.robinsonliste.de/	Nachhaltiger, weniger Material- verbrauch, spart Ressourcen
	Prinzip „Reparatur vor Neukauf" in allen Bereichen etablieren	Nachhaltiger
	Konzept für die Weiternutzung alter Geräte entwickeln: Weitergabe älterer Laptops, Beamer, etc.	Nachhaltiger
**Lebensmittel und Catering**	Reduktion des Konsums von Fleischprodukten	Spart Ressourcen
	Regionales und saisonales Obst und Gemüse verwenden	Spart CO_2
	Einsatz von Bio- und Fairtrade-Produkten	Nachhaltiger
	Nutzung von Mehrweggeschirr	Vermeidet Abfall
**Ressourcen allgemein**	Materialverschwendung vermeiden	Spart Ressourcen
	Recyclingpapier und Briefumschläge benutzen	Spart 70 % Wasser und 60 % Energie in der Herstellung gegenüber Frisch-faserpapier
	Elektronische Dokumente und digitale Datenerhebung nutzen, wo möglich auf Ausdrucken verzichten	Spart Papier und Kosten
	Gemeinschaftsnutzung von Technik/Geräten/Räumen, um Leerlauf zu vermeiden	Spart Ressourcen, reduziert Verbrauch

Bereiche	Was	Nutzen
**Wasser**	Bewässerung von Außenanlagen mit Regenwasser	Reduzierter Verbrauch
	Kurzspültaste in Toiletten nutzen	Reduzierter Verbrauch
	Wassersparaufsätze einbauen	Reduzierter Verbrauch
**Beschaffung**	Bei der Anschaffung neuer Elektrogeräte auf Nachhaltigkeit und Energieeffizienz achten:<br>– Langlebige Geräte, die repariert werden können<br>– Tonerkartuschen, die nachgefüllt werden können	Geringerer Stromverbrauch, spart Energie, Kosten und Müll
	Tintenstrahldrucker mit nachfüllbaren Tanks nutzen	Spart Materialverbrauch und Kosten
	Einsatz von biologisch abbaubaren Seifen und Putzmitteln	Nachhaltiger
**Geräte und Bürobedarf**	Etablierung von Richtlinien für Nachhaltigkeit bei Beschaffung/Einkauf, z.B.:<br>– Schadstoffarme Geräte beschaffen<br>– Reparaturverträge abschließen<br>– Energiesparende Materialien bevorzugen<br>– Wo möglich, biologisch abbaubare Materialien beschaffen<br>– Auf Gütesiegel achten	Verankertes Nachhaltigkeitskonzept
	Bei Kooperation mit externen Partnern bzw. Unternehmen auf Umsetzung von Nachhaltigkeit des Unternehmens achten	Gemeinsam nachhaltiger
**Immobilien/ Gebäude**	Dämmung und Verschattung statt Klimaanlage	Reduzierter Verbrauch
	Hitzeschutzpläne etablieren	Prävention
	Analyse von Einsparpotentialen:<br>– Ggf. energetische Sanierung von Bestandsbauten<br>– Bei Neubauten auf hohe Energieeffizienzstandards setzen	Spart Ressourcen, reduziert Verbrauch
	Analyse von Raumnutzung – wie können Gebäude/Räume noch effizienter genutzt werden?	Spart Ressourcen
	Garantie für Zugang zu verschatteten Gärten mit Bäumen statt nur „Freiflächen“ bei Unterbringung nach PsychKG[3]	Prävention; spezifisch für Kliniken
**Klimaschutz-beauftragte**	Klimaschutzbeauftragte Person benennen, jährliche Berichterstattung	Strategischer und strukturierter Klimaschutzansatz
**Organisations-strukturen der Einrichtung und Kommunikation**	Unternehmenskommunikation: Marketing für Nachhaltigkeit	Fördert Nachhaltigkeit
	Nachhaltige Bank und Versicherung	Fördert Nachhaltigkeit
	Technik-Führungen für Beschäftigte	Fördert Nachhaltigkeit

3 Gesetz über Hilfen und Schutzmaßnahmen bei psychischen Krankheiten (PsychKG) Berlin, § 18 Einrichtungen, Gliederung und Ausstattung: „(4) Die Einrichtungen müssen über die Voraussetzungen zur Durchführung von freiheitsentziehenden Maßnahmen verfügen. Gesicherte Freiflächen sind in angemessener Größe vorzuhalten und zur Freizeitgestaltung zur Verfügung zu stellen. […] Bei der Durchführung von freiheitsentziehenden Maßnahmen ist das Entweichen der unterge- brachten Personen durch geeignete Maßnahmen zu verhindern.“

Bereiche	Was	Nutzen
**Schulungen**	Schulungen und Informationen für die Mitarbeitenden<br>Jede Berufsgruppe ansprechen, informieren Sie alle Stakeholder<br><br>Das Management interessiert z.B.<br>– Finanzieller Nutzen<br>– Abgrenzung zu anderen Kliniken<br>– Wertsteigerung der Immobilie durch eine Maßnahme<br><br>Das Personal interessiert z.B.<br>– In einem angenehmen Umfeld zu arbeiten<br>– Persönlicher Nutzen für zuhause<br>– Bei einer Arbeitsstelle mit gutem Ruf tätig zu sein	Fördert Nachhaltigkeit
	Informationen vermitteln über Aufkleber und Plakate	Sensibilisierung für Nachhaltigkeit
**Spezifisch für Kliniken und Praxen**		
**Digitalisierung**	EMRAM-Score erhöhen (*Electronical Medical Record Adoption Model*)	Systematischer Klimaschutz durch Digitalisierung, weniger Materialien-gebrauch sowie kommunikative/ sensibilisierende Wirkung
	Mehr digitale Angebote z. B. *doctolib*, Online-Zugangsplattformen für Patienten, Patienten-Apps und Apps für medizinisches Personal, stärkere digitale Erfassung von Gesundheitsdaten	Schnellere Prozessabläufe, bessere interne und externe Vernetzung, verbesserte Versorgung
	Mehr Nutzung von Telepsychiatrie und -psychotherapie	Weniger Energieverbrauch (Fahrten, Räume)
	Elektronische Patientenakte (EPA) nutzen	Verringerung der Papierlast, schnellere Prozessabläufe, bessere interne und externe Vernetzung, verbesserte Versorgung
**Terminstrukturie-rung**	Vermeidung von Leerterminen (*not attending*): Terminstrukturierung/Therapiepläne verbessern	Spart Ressourcen
**Nutzerinnen-/ Nutzerbeteiligung**	Partizipation der Nutzenden, um gemeinsam Maßnahmen herauszufinden, zu diskutieren und umzusetzen: Lassen Sie die Nutzenden mitmachen und stimmen Sie mit ihnen ab, wie Energiesparmaßnahmen gelingen, ohne die Arbeitsabläufe zu erschweren. Je einfacher und nutzerfreundlicher die Lösung, desto schneller wird sie angenommen.	Größere Akzeptanz und *ownership*
**Vorbereitung auf Extremwetterlagen**	Hitzeschutzpläne/Leitlinien für Kliniken und Praxen, siehe auch „Handlungsempfehlungen für die Erstellung von Hitzeaktionsplänen zum Schutz der menschlichen Gesundheit“4 des Umweltbundesamtes	Spart Ressourcen, wirkt präventiv
**Spezielle Angebote**	Klimasprechstunde: Sprechstunde für klimarelevante Fragen von Patientinnen und Patienten und zur Sensibilisierung für das Thema „Klimaschutz ist Gesundheitsschutz“, Handlungsmöglichkeiten aufzeigen	Sensibilisierung für das Thema „Klimaschutz ist Gesundheitsschutz“, bietet Handlungsmöglichkeiten an
**Zertifizierung**	Zertifizierung der Abteilung/Praxis anstreben, DGPPN-Umweltzertifikat entwickeln, an bestehende Zero-Emissions-Initiativen anschließen	Fördert Nachhaltigkeit

Bereiche	Was	Nutzen
**Spezifisch für Forschungseinrichtungen**		
**Leitungsebene**	Nachhaltigkeit in Leitbild von Forschungseinrichtungen verankern	Selbstverpflichtung zu Nachhaltigkeit
	Konkrete Nachhaltigkeitsziele festlegen und Richtlinien für verschiedene Forschungs- und Verwaltungsbereiche etablieren	Selbstverpflichtung zu Nachhaltigkeit
	Bereitstellung finanzieller und personeller Unterstützung, um Nachhaltigkeitsziele umzusetzen	Fördert Nachhaltigkeit
	Einforderung von Nachhaltigkeitskonzepten durch Forschungsförderinstitutionen	Fördert Nachhaltigkeit
	Anreizsystem: Etablierung von Forschungspreisen für herausragende nachhaltige Forschung auf dem Gebiet „Klima und Psyche“	Fördert Forschung, generiert Wissen
**Projektebene**	Individuelle Nachhaltigkeitskonzepte je Forschungsprojekt entwickeln	Spart Ressourcen
	Wirkungen eigener Forschungsarbeiten reflektieren: Welche günstigen und ungünstigen langfristigen Effekte hinsichtlich Energie-/Ressourcenverbrauch sind zu erwarten?	Spart Ressourcen
	Synergieeffekte durch Vernetzung nutzen	Spart Ressourcen
	Open-Access-Publikationen fördern	Spart Ressourcen
**Kommunikation**	Informationsvermittlung/Sensibilisierung und Ermöglichung von Kompetenzerweiterungen zum Thema „Nachhaltigkeit“	Fördert Nachhaltigkeit, spart Ressourcen
	Anreizsystem für Umsetzung nachhaltigen Verhaltens etablieren	Fördert Nachhaltigkeit
	Regelmäßige Berichterstattung von Forschungsorganisationen zur eigenen nachhaltigen Forschung	Fördert Nachhaltigkeit

4 https://www.bmuv.de/fileadmin/Daten_BMU/Download_PDF/Klimaschutz/ hap_handlungsempfehlungen_bf.pdf

A3 Psychiatrisch-psychotherapeutische Behandlungskette optimieren

Bereiche	Was	Nutzen
**Deckung von Grundbedürfnissen als Voraussetzung zur Verhinderung von Morbidität und Behandlungs-bedarf**	– Förderung selbständigen Wohnens<br><br>– Aktive Verhinderung sozialer Isolation: „*Befriending*“, Förderung von Kontakten von Menschen mit psychischen Problemen in der Gemeinde<br><br>– Förderung von Arbeit und Beschäftigung, z.B. via *Supported Employment*: aktive Zusammenarbeit mit Arbeitsförderung und Wirtschaft, Beteiligung an Kooperativen<br><br>– *Green and blue Space*: *Awareness-raising* und Beteiligung an Initiativen zur Schaffung von Erholungsräumen in Städten und Gemeinden	*Housing First* z. B.: weniger Obdachlosigkeit, weniger vollständige Heimversorgung<br><br>Weniger ersatzweise (Fehl-)Inanspruchnahme von psychiatrischen Diensten<br><br>Größere Lebenszufriedenheit und Partizipation, weniger Rückfälle und Inanspruchnahme von Diensten<br><br>Größere Lebenszufriedenheit
Empowerment **von Betroffenen**	– *Empowerment*<br>– Selbstsorge	Geringere Abhängigkeit vom Versorgungssystem
Social prescribing	– Reduktion nicht-pharmakologischer Bewältigung emotionaler, sozialer und praktischer Probleme als Teil von *Empowerment*	– Spart Ressourcen (weniger Inanspruchnahme/ Verbrauch von Medikamenten, z. B. sedierende Medikation bei Schlafstörungen)
De-prescribing **und Optimierung der psychophar-makologischen Medikation**	– Reduktion von Polypharmazie und medizinisch begleitetes Umstellen/Abdosieren/Absetzen von Medikation mit negativem Nutzen-Risiko-Profil [1] (z. B. Analgetika und sedierende Medikation mit erhöhtem Sturzrisiko oder erhöhtem Interaktionsrisiko; z. B. basierend auf der *Choosing wisely“*-Initiative [2]<br>– Regelmäßige Arzneimittel-Reviews in Kliniken (zusammen mit Pharmakologinnen/Pharmakologen) [3]	Spart Ressourcen durch weniger Inanspruchnahme/Verbrauch von Medikamenten
**Ambulantisierung der Behandlung**	– Eine Reduktion stationärer psychiatrischer Dienste durch mehr ambulante Behandlung kann zu geringerem Ressourcenverbrauch beitragen, wenn nicht eine Verlagerung des Verbrauchs ins ambulante System oder höhere Mobilitätskosten durch aufsuchende Behandlung (z. B. durch mehr Autofahrten) auftritt, die Versorgung durch den nicht-stationären Bereich gewährleistet ist und die Versorgung nicht schlechter wird	Reduktion der Anzahl von stationären Plätzen ist klimaschonend und spart Ressourcen

A4 Neue Behandlungsangebote schaffen – Anpassung an mögliche Veränderungen des psychiatrischen Handlungsbedarfs und Diagnosespektrums im Rahmen des Klimawandels

Bereiche	Was	Nutzen
**Anpassung des Angebots**	Anpassung an häufigeres Auftreten von psychischen Krisen durch Katastrophenereignisse (Hitze, Überschwemmungen): (Klima-)Angsterkrankungen, Traumafolgestörungen,<br>soziale Isolation<br><br>Aufbau von Spezialambulanzen	Verhinderung einer Chronifizierung klimabedingter Veränderung psychischer Gesundheit
**Kooperation mit somatischen medizinischen Fächern**	Bessere konsiliarisch-somatische Gesundheitsversorgung bei schlechterer somatischer Gesundheit vulnerabler psychisch erkrankter Patientengruppen (schwer psychisch Kranke, alte Menschen, Kinder)	Verringerung somatischer Morbidität mit dem Risiko von Folgeerkrankungen
**Soziale Integration psychiatrisch-psychotherapeutischer Arbeit**	Förderung sozialer Netzwerke und unterstützender sozialer Beziehungen durch Psychiaterinnen und Psychiater sowie Psychotherapeutinnen und -therapeuten (zusätzlich zur indivi- duellen Therapie), um einen zu isoliert individuellen Umgang mit Klimaauswirkungen, Klimaängsten und angstbedingte psychische Morbidität zu vermeiden oder abzuschwächen [4]	Förderung kollektiver Resilienz als Ressource der sozialen Gemeinschaft
**Vulnerable Gruppen**	Kultursensible Angebote und Sprachmittlung gewährleisten, Unter- oder Fehl-Versorgung von Personen mit Migrationshintergrund oder geflüchteten Personen vermeiden. Ein Anstieg von Migration ist im Rahmen des Klimawandels möglich.	Versorgung von Personen mit Migrationshintergrund oder geflüchteten Personen

A5 Strategien einer nachhaltigen Forschung

Bereiche	Was	Nutzen
**Institutionelle Verankerung/ Governance**	Verankerung des Nachhaltigkeitsgedankens im Leitbild	Sensibilisierung für Nachhaltigkeit
	Verankerung des Prinzips der Nachhaltigkeit im übergeordneten Management von Forschungsorganisationen: Analyse (Identifizierung von Emissionen und Einsparpotentialen), konkrete Zielsetzung und Strategieauswahl	Implementierung (siehe Anhang 6), Monitoring (definierte Klima-Indikatoren zur Prozessüberwachung)
	Berichterstattung: Aufklärung über erreichte Nachhaltigkeitsziele im Jahresbericht oder Etablierung eines eigenen Klimaberichts	Berichterstattung nach innen und außen
**Forschungs-finanzierung und -förderung**	Einforderung von Nachhaltigkeitskonzepten durch Forschungs-förderinstitutionen (z. B. Drittmittelgeber)	Sensibilisierung für Nachhaltigkeit und Ressourcenschonung in der Forschung
	Förderung von Forschungsprojekten, die sich spezifisch mit den Auswirkungen des Klimas auf die Psyche sowie relevanten Adaptions- und Mitigationsmaßnahmen beschäftigen	Wissen generieren und neue Handlungsmöglichkeiten aufzeigen
	Anreizsystem: Etablierung von Forschungspreisen für herausragende nachhaltige Forschung auf dem Gebiet „Klima und Psyche“	
**Gestaltung eines nachhaltigen Forschungs-prozesses**	Bekennung zu Nachhaltigkeit formulieren und ein individuelles Nachhaltigkeitskonzept für Forschungsprojekte erstellen	Sensibilisierung für Ressourcenverbrauch
	Ressourcenverbrauch des Projekts analysieren und überwachen	
	Einsparpotentiale identifizieren, hierzu zählen insbesondere: Dienstreisen/Mobilität, gemeinschaftliche Nutzung von Arbeitsplätzen/Geräten/Materialien etc.	
	Wirkungen der Forschungsarbeiten reflektieren: Welche günstigen und auch ungünstigen langfristigen Effekte hinsichtlich Energie-/Ressourcenverbrauch sind zu erwarten?	

Bereiche	Was	Nutzen
	Reduzierung verschwenderischer Aktivitäten, wo möglich (*wasteful activities*):<br>– Materialverschwendung vermeiden<br>– Umweltschonende Entsorgung von Geräten/Materialien im Laufe und nach Abschluss des Forschungsprojektes<br>– Insuffiziente Arbeitsprozesse durch fehlenden Projektverantwortlichkeiten vermeiden<br>– Methodische Fehler in der Studienführung vermeiden, die Studienergebnisse nur stark limitiert interpretierbar machen<br>– Siehe auch Anhang 6	Sensibilisierung für Ressourcen-verbrauch
	Ausrichtung an *Sustainable Development Goals* der UN	
	Open Access publizieren	
**Nutzung von Synergieeffekten durch Vernetzung**	Wissenstransfer insbesondere zu Methodik, Praxiskompetenzen, Antragstellung etc.	Spart Ressourcen und Zeit, besserer Wissenstransfer
	Wissenschaftslandschaft: prüfen, wer arbeitet noch an demselben Thema oder mit ähnlicher Methodik; Schaffung vergleichbarer Daten und Methoden, damit Ergebnisse über verschiedene Projekte und Institute besser vergleichbar sind und die empirische Evidenz somit erhöht wird	
	Forschungspraxis: Können Ressourcen gemeinsam genutzt/verwaltet werden?	

A6 Standards und Richtlinien für nachhaltige Forschung

**Prinzipien Nachhaltigkeit**	
UN Sustainable Development Goals	https://sdgs.un.org/goals
Responsible Research and Innovation der EU-Strategie Horizon 2020	https://ec.europa.eu/programmes/horizon2020/en/h2020-section/responsible-research-innovation
Deutsche Nachhaltigkeitsstrategie der Bundesregierung	https://www.bundesregierung.de/breg-de/themen/nachhaltigkeitspolitik/deutsche-nachhaltigkeitsstrategie-318846
**Guidelines für Forschungsaktivitäten**	
Fraunhofer Klimaneutral 2030	https://www.fraunhofer.de/content/dam/zv/de/ueber-fraunhofer/wissenschaftspolitik/Positionen/politikpapiere-btw21/Politik-Papier_Klimaneutral%202030%20WEB.pdf
Handlungsempfehlungen und Checklisten der Deutschen Gesellschaft für Nachhaltigkeit an Hochschulen e. V. (DG HOCH^{N})	https://www.dg-hochn.de/dokumente
Handreichung des LeNa-Projektes zum Nachhaltigkeits-management außeruniversitärer Forschungseinrichtungen	https://www.nachhaltig-forschen.de/startseite/
GoGreenGuide Uni Dresden	http://2017.igem.org/wiki/images/6/60/T--TU_Dresden--GoGreenGuide.pdf
Harvard University Green Labs Guide	https://green.harvard.edu/programs/green-labs

A7 Inhalte einer Aus-, Fort- und Weiterbildung zu „Klimawandel und Psychiatrie/Psychotherapie“

**Grundlagen des menschengemachten Klimawandels**	
Wissenschaftliche Grundlagen	– Menschengemachter Treibhauseffekt<br>– CO_2-Fußabdruck
Planetare Gesundheit	– Konzepte, Begriffe
Einfluss des Klimawandels auf die menschliche Gesundheit	– Direkte und indirekte Einflüsse
Klimagerechtigkeit	– Aufklärung über gesundheitliche Ungleichheit und soziale Determinanten von (psychischer) Gesundheit<br>– Bedeutung von Klimagerechtigkeit für sozialen Zusammen- halt innerhalb von Gesellschaften und Weltfrieden
Klimawandel und Genderfragen	– Geschlechtsspezifische Auswirkungen des Klimawandels
**Psychiatrische Aspekte des Klimawandels**	
Psychische Folgen der Klima- und Ökokrise	– Allgemeine Grundlagen<br>– Spezifische Folgen, z.B. psychische Folgen von Extremwetterereignissen
Hitze und ihre Auswirkungen	– Aggressivität<br>– Wirksamkeit von Medikamenten
Neu beschriebene Formen psychischer Belastung	– Klimaangst und andere Klimagefühle<br>– Solastalgie
Psychische Mechanismen der Verarbeitung	– Psychodynamische, kognitiv-behaviorale, soziologische, evolutionäre Erklärungsmodelle
Handlungsleitlinien für stationäre, teilstationäre und ambulante Behandlungen	– Schwerpunktthemen wie Hitze, Bewegung und Ernährung
Resilienz in Zeiten des Klimawandels	– Strategien zur Vermeidung von Burnout und Resignation beim klimabewussten Handeln
**Rolle des Gesundheitswesens und seiner Mitarbeitenden**	
Aktivismus	– Geschichte des Aktivismus in der Medizin – Rudolf Virchow, Anti-Tabak-Kampagnen, Anti-Atomwaffen-Bewegung (IPPNW)<br>– Die besondere Rolle/Verantwortung der Psychiatrie was politische Repression betrifft (Geschichte der Psychiatrie – Menschenrechtsverletzungen und Zwangseinweisung von politischen Dissidenten)<br>– Die Rolle von Aktivismus für die psychische Gesundheit<br>– Die Zukunft von Klima-Aktivismus
Nachhaltigkeit im Gesundheitswesen	– CO_2-Abdruck von Medikamenten, NHS Klimaneutral bis 2040

**Rolle des Gesundheitswesens und seiner Mitarbeitenden**	
Prävention in Zeiten des Klimawandels	– *Public Mental Health* – zur sowohl vorbeugenden als auch intervenierenden Rolle der Psychiatrie (*Psychiatrist's role as preventionist as well as interventionists*)<br>– Individuelle Prävention
Mechanismen der Inaktivität und Beeinflussung	– Psychologische Mechanismen<br>– Gesellschaftliche Mechanismen<br>– Arbeit von Lobbyorganisationen
Erklärung der Klimakrise,<br>Psychiater als *change agents*	– Bedeutung der Klimakrise<br>– Rolle und Verantwortung der Medizin in Zeiten des Klimawandels
Ethische und juristische Fragen	– Rechtsprechung des Bundesverfassungsgerichts: Klimaschutz als Menschenrecht<br>– Gesunde Umwelt als Menschenrecht – wurde am 08.10.2021 vom UNHR Council anerkannt, UN appelliert an Staaten dies anzuerkennen<br>– Kinderrechte
Kommunikation im Angesicht der Klimakrise	– Umgang mit Skeptikern oder Leugnern des Klimawandels<br>– Wie erreicht man Menschen?

A8 Materialen, Ressourcen und Quellen für Aus-, Fort- und Weiterbildung

**Materialien aus Deutschland**	
Stellungnahme DGPPN	https://www.dgppn.de/_Resources/Persistent/40f00ff8b9f34c1f5467c182ef29a7e50463a739/2019-11-27_FIN_STN%20zu%20Auswirkungen%20Klimaveränderungen%20auf%20die%20psych.%20Gesundheit.pdf
Positionspapier BDP	https://www.bdp-verband.de/binaries/content/assets/ politische-positionen/2021/20211006_bdp_stellungnahme_ psychologie-und-klima.pdf
Leitfaden Planetare Gesundheit KLUG	https://www.klimawandel-gesundheit.de/wp-content/uploads/2022/01/Leitfaden-Planetary-Health-Lehre-2022_01.pdf
Planetary Health Academy	https://planetary-health-academy.de/
Scientists4future Präsentationssammlung	https://files.scientists4future.org/
**Materialien international**	
Royal College of Psychiatrists Positionspapier Mai 2021	https://www.rcpsych.ac.uk/docs/default-source/ improving-care/better-mh-policy/position-statements/ position-statement-ps03-21-climate-and-ecological- emergencies-2021.pdf?sfvrsn=281fb719_10
Royal College of Psychiatrists Eco-Anxiety Resources 2020	https://www.rcpsych.ac.uk/mental-health/parents-and- young-people/information-for-parents-and-carers/eco-distress---for-parents-and-carers<br><br>https://www.rcpsych.ac.uk/mental-health/parents-and- young-people/young-people/eco-distress---for-young-people
Royal College of Psychiatry Planetary health and sustainability committee	https://www.rcpsych.ac.uk/improving-care/working-sustainably/about-sustainability-in-mental-health-care
Association of Clinical Psychologists UK Positionspapiere	https://acpuk.org.uk/climate_change_statement/<br><br>https://acpuk.org.uk/acp-uk-rapid-response-to-report-on- impact-of-climate-breakdown-on-distress/
UCL Climate Hub	https://www.ucl.ac.uk/bartlett/news/2021/jul/climate-change-and-mental-health
Green Prescribing	https://sustainablehealthcare.org.uk/blog/sustainable-prescription-mental-health-care-green-social-prescribing

**Materialien international**	
Course in sustainable mental health care	https://sustainablehealthcare.org.uk/courses/sustainable-mental-healthcare
Courses in nature based practice Ecopsychology/Ecopsychotherapy UK	https://www.naturalacademy.org<br><br>https://circleofliferediscovery.com/education-and-outdoor-learning/certificate-in-nature-based-practice/<br><br>https://www.confer.uk.com/module/module-ecopsychotherapy.html
Planetary Health Alliance Resources	Planetary Health Education Framework: https://drive.google.com/file/d/1wg2zJnKj-wlGN5qK8EXC0Y2lqUwMUQNV/view
Mental Health Foundation UK	https://www.mentalhealth.org.uk/our-work/policy-and-advocacy/climate-change
United Nations Conference of the Parties (COP) Resources	https://unfccc.int/process/bodies/supreme-bodies/conference-of-the-parties-cop

**Literatur**	
Übersichtsarbeiten	Lawrance E, Thompson R, Fontana G et al. (2021) The impact of climate change on mental health and emotional wellbeing: current evidence and implications for policy and practice. [5]
Nachhaltige Psychiatrie	Monsell A, Krzanowski J, Page L et al. (2021) What mental health professionals and organisations should do to address climate change. [6]
Nachhaltiges Verschreiben	Cussans A, Harvey G, Kemple T et al. (2021) Interventions to Reduce the Environmental Impact of Medicines: A UK perspective. [7]
Eco-Anxiety/psychische Belastung	Panu P (2020) Anxiety and the Ecological Crisis: An Analysis of Eco-Anxiety and Climate Anxiety. [8]<br><br>Hickman C, Marks E, Pihkala P et al. (2021) Climate anxiety in children and young people and their beliefs about government responses to climate change: a global survey. [9]
Psychotherapie	Nikendei C (2020) Klima, Psyche und Psychotherapie: Kognitionspsychologische, psychodynamische und psychotraumatologische Betrachtung einer globalen Krise. [10]
Rahmenmodell für die Aufklärung über planetare Gesundheit	Guzmán CAF, Aguirre AA, Astle B et al. (2021) A framework to guide planetary health education. [11]

A9 Checkliste zur Organisation einer klimabewussten Aus-, Fort- und Weiterbildung

**Mobilität (An- und Abreise, Mobilität vor Ort)**
Der Veranstaltungsort ist vom/zum nächstgelegenen Bahnhof zügig und mit geringem Aufwand mit öffentlichen Verkehrsmitteln zu erreichen
Vorrangige Kommunikation einer klimaschonenden An- und Abreise
Informationen über die Anbindung an den Fernverkehr (Zug- oder Busverbindungen) und den lokalen Personennahverkehr mit Ankunftszeiten und Frequenzen
Sonstige Informationen werden genau dargestellt und vorrangig vor den Anreisemöglichkeiten mit dem Auto erklärt
Anreize, Belohnung und Unterstützung einer umweltfreundlichen An- und Abreise
Der Veranstalter motiviert alle Beteiligten zur umweltfreundlichen An- und Abreise und/oder unter-stützt sie aktiv dabei. Beispielsweise durch vergünstigte Teilnahmegebühren bei Anreise mit öffentlichen Verkehrsmitteln
Allen Beteiligten wird bereits bei Bewerbung der Veranstaltung, spätestens aber bei der Anmeldung die Möglichkeit zur Nutzung öffentlicher Verkehrsmittel (Fahrpläne, Haltestellen etc.) oder anderer umweltfreundlicher Alternativen vor Ort (Fahrradverleih etc.) kommuniziert
Side-Events, Ausflüge etc. sind so organisiert, dass sie von allen Beteiligten zu Fuß, mit dem Fahrrad oder mit öffentlichen Verkehrsmitteln erreichbar sind oder mit einem Sammel-Shuttledienst durchgeführt werden
Allen Beteiligten wird die Möglichkeit geboten, ein Ticket für den ÖPNV vor Ort über die Organisatoren zu erwerben
Information über Treibhausgas-Kompensation
Die Veranstaltungszeiten (Beginn und Ende) werden auf die Fahrpläne (Ankunfts- und Abfahrtszeiten) von Zug und Bus (Regional- und/oder Fernverkehr) abgestimmt
**Gestaltung der Fort- und Weiterbildung: Materialien**
Information aller Beteiligten über Abfallvermeidung und Abfalltrennung vor Ort
Ressourcenaufwand für Papier/Druck: Sämtliche veranstaltungsrelevanten Druckwerke (Einladungen, Dokumentationen etc.) sind nach dem Prinzip des minimalen Ressourcenaufwands angefertigt – geringe Auflage, kleines Druckformat, doppelseitige Kopien, Ersatz durch elektronische Datenträger, Mail-Services, Internet, Apps etc.
Papierqualität von Druckwerken für die Veranstaltung: Das verwendete Papier bei externen Druckaufträgen (Prospekte, Briefpapier, Programme, Kuverts etc.) trägt ein Umweltzeichen oder ist zu 100 % Recyclingpapier
Keine Give-Aways, die große Abfallmengen oder umweltschädliche Abfälle verursachen (Einweggetränkeverpackungen, Produkte mit Batterien oder Akkus, Tagungsmappen etc.)

**Gestaltung der Fort- und Weiterbildung: Gastronomie**
Es werden bevorzugt Mehrwegbecher, Mehrweggeschirr (Teller, Schüsseln) und Mehrwegbesteck verwendet
Einkauf von Getränken ausschließlich in Groß- und/oder Mehrweggebinden und Ausschank aus diesen
Keine Verwendung von Portionsmaschinen mit Einweg-Einzelportionsverpackungen für Kaffee oder Tee
Hauptzutaten der Gerichte sind nach Möglichkeit saisonal, regional, biologisch und/oder fair gehandelte Produkte, gekennzeichnet mit entsprechender Zertifizierung
Es werden keine aus Sicht des Tier- und Artenschutzes bedenklichen Lebensmittel verwendet
Mindestens ein vegetarisches oder veganes Gericht wird angeboten
**Kommunikation**
Eine qualifizierte Ansprechperson („Green Meeting-/Green-Event-Beauftragte") wird bekannt gegeben und steht allen Beteiligten und der Öffentlichkeit für Anfragen zur Verfügung: vor, während (vor Ort) und nach der Veranstaltung
Die Veranstaltenden befragen die Teilnehmenden, Besucher und/oder das Publikum mit geeigneten Mitteln zu ihrer Meinung über die Green-Meeting-/Green-Event-Maßnahmen oder geben ihnen auf andere Weise eine Möglichkeit für Rückmeldungen (schriftliche Fragebögen, Feedbackfunktion online). Die Ergebnisse der Befragung werden ausgewertet.

A10 Aktionsplan für eine nachhaltige Fachgesellschaft

Die Fachgesellschaft macht sich auf den Weg in Richtung Klimaneutralität. Die DGPPN-Task- Force „Klima und Psyche“ hat die Kernbereiche der Fachgesellschaft hinsichtlich möglicher Maßnahmen auf verschiedenen Handlungsebenen überprüft. Daraufhin wurden entsprechende Bewegungs- und Handlungsspielräume definiert und konkrete Maßnahmen vom DGPPN-Vor- stand beschlossen. Eine Auswahl der Maßnahmen wird im Folgenden dargestellt.

Warum ist ein Klima-Aktionsplan für die Fachgesellschaft notwendig?
Die Beeinträchtigung des Lebens durch Klima- und Umweltzerstörung ist eine Krise, die aus Sicht der DGPPN eine noch nie dagewesene Bedrohung für die menschliche Gesundheit dar- stellt. Klimawandel, Umweltverschmutzung und der Verlust der biologischen Vielfalt haben besondere Auswirkungen auf die psychische Gesundheit und betreffen in besonderem Maße vulnerable Gruppen wie Menschen mit psychischen Erkrankungen und Kinder. Die DGPPN ist ihrem Selbstverständnis nach eine Organisation, die aktiv an der Bewältigung der Klima- und ökologischen Krise arbeitet und gleichzeitig die Nachhaltigkeit im Gesundheitswesen fördert.

Aktionsfelder des DGPPN-Aktionsplans für eine klimaneutrale Arbeit der Fachgesellschaft

Bewusstseinsbildung	DGPPN Kongress
Interessenvertretung	Finanzwirtschaft
Forschungsförderung	DGPPN-Geschäftsstelle

Bewusstseinsbildung

Ausgangssituation

Neben den unmittelbaren Auswirkungen, die Naturkatastrophen und extreme Wetterbedingungen im Rahmen des Klimawandels auf die psychische Gesundheit von Menschen haben können, wirkt sich die Veränderung von Natur und Umwelt auch über indirekte Wege, d. h. über soziale, wirtschaftliche oder anderweitig langfristige Prozesse ungünstig auf die Determinanten psychischer Gesundheit aus. Die Prävalenz von psychischen Erkrankungen, vor allem Depressionen, Angststörungen und posttraumatische Belastungsstörungen, steigt aufgrund der weitreichenden Auswirkungen des Klimawandels weltweit an. Der Zusammenhang von Klimaschutz und Gesundheitsschutz ist vielfach auch unter Medizinerinnen und Medizinern noch zu wenig bekannt. Hinzu kommt eine neue Form des *climate distress*, ein Gefühl der Hilflosigkeit, Sinnlosigkeit und eine Wahrnehmung, dass die Zukunft nicht lohnenswert ist.

Zielsetzung

Die in der Behandlung von Menschen mit psychischen Erkrankungen Tätigen sollen über die Zusammenhänge von Klima und Psyche informiert sein und sich der aus ihrer Expertise folgenden gesellschaftlichen Verantwortung bewusst werden. Dazu gehört sowohl die Aufklärung und Motivation zu klimafreundlicher Verhaltensänderung bei Patientinnen und Patienten, der Beitrag zu Resilienzförderung sowie der Umgang mit *climate distress* etc. Darüber hinaus soll auch die Öffentlichkeit laufend über neue Erkenntnisse zu diesen Zusammenhängen informiert werden.

Ausgewählte Maßnahmen

Veranstaltungen zum Thema Klima und psychische Erkrankungen

Die DGPPN wird über den Zusammenhang von Klimawandel und psychischen Erkrankungen in verschiedenen Formaten und Veranstaltungen informieren. Beispielsweise soll der Kompaktkurs für die Facharztprüfung für Psychiatrie und Psychotherapie um ein entsprechendes Modul erweitert werden. Weiterhin soll der DGPPN Kongress als Plattform für Symposien und Diskussionsforen zum Thema zur Verfügung stehen und den Austausch fördern. Zwischenziele auf dem Weg zum klimaneutralen Kongress ab 2030 sollen definiert, dokumentiert und das Erreichen regelmäßig kommuniziert werden, um als Modellprojekt mit Vorbildcharakter ein Zeichen zu setzen.
– Zeitrahmen: ab November 2022, regelmäßig

Veröffentlichungen zum Thema Klima und psychische Erkrankungen

Verschiedene Publikationen sollen das Problembewusstsein der im Bereich Psychiatrie und Psychotherapie Tätigen schärfen und Hilfestellungen geben. Neben dem vorliegenden Positionspapier inklusive Handlungsempfehlungen ist eine öffentliche „Berliner Erklärung“ zu den Auswirkungen des Klimawandels auf die psychische Gesundheit geplant. Das DGPPN-Magazin *Psyche im Fokus* wird ebenfalls regelmäßig und niedrigschwellig informieren. Weitere Informationsangebote sollen entwickelt werden.
– Zeitrahmen: Umsetzung bis März 2023

Interessenvertretung

Ausgangssituation

Die DGPPN bekennt sich im Einklang mit dem Deutschen Ärztetag im November 2021 zur ärztlichen Pflicht, die „Auswirkungen des Klimawandels klar zu benennen, die gesundheitliche Bedrohung durch den Klimawandel aufzuzeigen, Gegenmaßnahmen einzufordern und mit dazu beizutragen, dass sich das Gesundheitssystem auf die Bewältigung der Folgen des Klimawandels vorbereitet und bei jeglichem Handeln zum Wohle der Gesundheit klimaschädliche Auswirkungen vermeidet.“ [5]

---

5 Bundesärztekammer (2021) 125. Deutscher Ärztetag 2021 – Beschlussprotokoll. Verfügbar unter: https://www.bundesaerztekammer.de/aerztetag/aerztetage-der-vorjahre/125-daet-2021-in-berlin

Zielsetzung

Die politischen Entscheidungsträgerinnen und -träger sollen über die Auswirkungen des Klimawandels auf die psychische Gesundheit aufgeklärt werden. Speziell die Vulnerabilität von Menschen mit psychischen Erkrankungen soll wahrgenommen und es soll auf entsprechende Schutzmaßnahmen gedrängt werden (z. B. Finanzierung kommunaler Hitzeschutzmaßnahmen).

Ausgewählte Maßnahmen

Lobbyarbeit des Vorstands und der Geschäftsstelle
Der Vorstand und die Geschäftsstelle werden ihre Möglichkeiten der Lobbyarbeit für Klimaschutz nutzen und den Zusammenhang mit dem Gesundheitsschutz propagieren, z. B. durch Platzierung des Positionspapiers „Klima und Psyche“, Ansprache von Abgeordneten und Stellungnahmen zu Gesetzgebungsprozessen. Speziell bei Stakeholdern in der Gesundheitspolitik soll über diese Maßnahme ein Problembewusstsein für die besonderen Bedürfnisse von psychisch Erkrankten in Zusammenhang mit dem Klimawandel geschaffen werden.
– Zeitrahmen: ab sofort, regelmäßig

Forschungsförderung

Ausgangssituation

Zu erwarten ist, dass mit steigender Erderwärmung die Häufigkeit von Extremwetterlagen zunimmt und mit den indirekten Folgen des Klimawandels auch die psychische Belastung für größere Teile der Weltbevölkerung zunehmen wird. Neben Umweltschutzmaßnahmen sind gezielte Prävention und adäquate psychosoziale Interventionen notwendig, um das Ausmaß des psychischen Leidensdrucks der Weltbevölkerung zu begrenzen.

Zielsetzung

Das Forschungsfeld zu Zusammenhängen zwischen Klimawandel und psychischer Gesundheit ist relativ jung und es sind noch viele Fragen unbeantwortet. Um Antworten auf die aktuellen und zukünftigen Herausforderungen in der Gesundheitsversorgung zu finden, soll entsprechende Forschung finanziell gefördert werden.

Ausgewählte Maßnahmen

Ausschreibung von Forschungsgeldern
Die DGPPN stellt bis zu 150.000 Euro zzgl. USt. für Arbeiten zu Zusammenhängen zwischen Klimawandel und psychischer Gesundheit sowie zu den Auswirkungen auf die psychiatrische Versorgung zur Verfügung.
– Zeitraum: Die Ausschreibung soll 2023 veröffentlicht werden.

DGPPN Kongress

Ausgangssituation

Der DGPPN Kongress findet jährlich an vier Tagen im CityCube Berlin mit bis zu 9.000 Besuche- rinnen und Besuchern statt. In den letzten Jahren hat sich in der Kongressorganisation bereits eine Kultur der Nachhaltigkeit durchgesetzt.

Zielsetzung

Der DGPPN Kongress soll ab 2030 möglichst klimaneutral abgehalten werden. Alle im Rahmen des Kongresses entstehenden Emissionen, die zu diesem Zeitpunkt nicht vermieden werden können, sollen durch Kompensationszahlungen ausgeglichen werden. Unterstützt werden dazu Projekte in den Bereichen „Erneuerbare Energien" und „Energieeffizienz", welche nachweislich Klimagase einsparen, die andernfalls entstanden wären, und anhand des *CDM-Gold-Standards* zertifiziert sind. Darüber hinaus soll die Förderung der Projekte einen Nutzen für die Menschen vor Ort und eine Entlastung der lokalen Umwelt mit sich bringen.

Ausgewählte Maßnahmen

Klimaneutrale Dienstleister beauftragen
Das CO_2-Einsparungspotential des Kongresses soll ausgeschöpft werden. Dienstleister und Aussteller aus der Industrie sollen entsprechend ausgewählt und zu nachhaltigem Handeln ver- pflichtet werden. Der aktuelle Messebauer des DGPPN-Stands arbeitet bereits klimaschonend. Zudem werden Aufbauten und Beschilderungen eingelagert und Leihmöbel verwendet.
– Zeitrahmen: fortlaufend

Umstellung auf Mehrweg-Trinkflaschen
Zur Müllvermeidung sollen Einweg-Trinkbecher abgeschafft und durch ein System von Mehr- weg-Trinkflaschen ersetzt werden. Hierzu werden wiederverwendbare Pfand-Wasserflaschen mit DGPPN-Logo vergeben, die an Stationen nachgefüllt werden können. Außerdem werden die Kongressteilnehmenden aufgerufen, eigene wiederbefüllbare Flaschen mitzubringen.
– Zeitrahmen: seit 2019 fortlaufend

Einschränkung des Druckprogramms
Im Sinne der Ressourcenschonung soll das gedruckte Programm zum Kongress sukzessive auf weniger Seiten dargestellt und langfristig abgeschafft werden. Das Programm soll dann ausschließlich online per Webportal und App verfügbar sein.
– Zeitrahmen: seit 2019, seitdem fortlaufend

Finanzwirtschaft der Fachgesellschaft

Ausgangssituation

Die DGPPN ist ein eingetragener Verein, finanziert aus Mitgliedsbeiträgen. Die Ein- und Ausgaben werden derzeit auf einem Geschäftskonto bei der HypoVereinsbank geführt. Viele konventionelle Banken stehen wegen Investitionen in Rüstungsgeschäfte, Atom- und Kohlestrom, Nahrungsmittelspekulation und Geschäftsbeziehungen mit fragwürdigen Unternehmen und korrupten Regimes in der Kritik. Das Vermögen der *Stiftung Seelische Gesundheit* wird vom Stifterverband treuhändisch verwaltet und ist in Fonds angelegt.

Zielsetzung

Das Vermögen und die Geldflüsse der DGPPN sollen nachhaltig und im Sinne des Klimaschutzes verantwortlich gestaltet werden.

Ausgewählte Maßnahmen

Nachhaltige Bank für Geschäftskonto und Anlagen
Banken, die nach ethischen und nachhaltigen Kriterien wirtschaften, spekulieren nicht mit Nahrungsmitteln und bremsen nicht die Energiewende, sie unterstützen keine Umweltzerstörung und arbeiten transparent. Daher soll der Umzug des DGPPN-Geschäftskontos zu einer nach- haltigen Bank geprüft werden, um einen in diesem Sinne verantwortungsvollen Umgang mit den Beiträgen der Mitglieder zu gewährleisten. Aktuelle und zukünftige Anlagen sollen auf Nachhaltigkeit geprüft werden.
– Zeitrahmen: wird geprüft

Nachhaltige Versicherungen
Versicherungen können Versicherungsbeiträge auf nachhaltige Weise anlegen. Solche Versicherungen unterstützen im Schadenfall regionale Unternehmen und finanzieren den nachhaltigen Ersatz (z. B. Reparatur vor Neukauf). Der Wechsel der laufenden Versicherungen (Haftpflicht, D&O) zu nachhaltig arbeitenden Anbietern soll den Klimaschutz direkt und indirekt fördern.
– Zeitrahmen: schnellstmögliche Umsetzung

DGPPN-Geschäftsstelle

Ausgangssituation

Die DGPPN-Geschäftsstelle mit 21 Angestellten befindet sich seit Januar 2022 in gemieteten Gewerberäumen im 1. OG in der Reinhardtstraße 29, 10117 Berlin. Die Geschäftsstelle umfasst neben einem Konferenzraum sieben Büros, einen Aufenthaltsraum, eine Küchenzeile, drei WCs sowie einen Server-/Materialraum (insgesamt ca. 400 m2). Geschäftsstellenleitung sowie die Mitarbeitenden sind schon seit einigen Jahren für das Thema Nachhaltigkeit sensibilisiert. Viele Maßnahmen zur CO_2-Reduktion wurden in den letzten Jahren bereits ergriffen. Dazu gehört zum Beispiel, dass die Geschäftsstelle ausschließlich Strom aus erneuerbaren Energien bezieht und damit die gesamte IT und der Server gespeist werden. Auch das Hosting der Webseiten basiert auf Ökostrom. Weiterhin werden ÖPNV-Tickets für Mitarbeitende finanziell unterstützt („BVG-Firmenticket“). Geschäftsreisen werden, wenn möglich, mit der Bahn realisiert. Bei der Bestellung von Lebensmitteln und Caterings wird auf Regionalität und Saisonalität geachtet. Milch und Kaffee werden als Bio- und Fairtrade-Produkte eingekauft. Die Ablage wird bereits weitestgehend papierfrei gestaltet.

Zielsetzung

Die DGPPN-Geschäftsstelle soll ab 2030 möglichst klimaneutral arbeiten.

Ausgewählte Maßnahmen

Klimabeauftragte benennen und Nachhaltigkeitsboard etablieren

Klimabeauftragte sensibilisieren die Kollegen, schlagen ihnen ressourcensparende Verhaltensänderungen vor und vereinfachen diese, wo möglich. Bei Neuanschaffungen/Prozessänderungen prüfen die Beauftragten die Klimaneutralität und suchen ggf. nach Alternativen. Die Klimabeauftragten bringen neue Vorschläge zur Emissionseinsparung ein und stehen bezüglich der Umsetzung beschlossener Maßnahmen (z. B. Einbau eines Wasserfilters und -sprudlers; Verschattung als Hitzeschutz) im regelmäßigen Austausch mit der Geschäftsstellenleitung.

– Zeitrahmen: ab Dezember 2022, dauerhafter Einsatz

Kompensation des CO_2-Abdrucks der Geschäftsstelle

Bei unvermeidbaren Emissionen soll der CO_2-Ausstoß kompensiert werden. Unterstützt werden dazu Projekte in den Bereichen „Erneuerbare Energien“ und „Energieeffizienz“, welche nach- weislich Klimagase einsparen, die andernfalls entstanden wären, und anhand des *CDM-Gold- Standards* zertifiziert sind. Darüber hinaus soll die Förderung der Projekte einen Nutzen für die Menschen vor Ort und eine Entlastung der lokalen Umwelt mit sich bringen.

– Zeitrahmen: ab Dezember 2022, jährlich

Literatur

1. Gupta S, Cahill J, Miller R (2019) Deprescribing in Psychiatry, 1. Aufl. https://doi.org/10.1093/med/9780190654818.001.0001

2. Maughan D, James A (2017) Diagnosis and treatment: Are psychiatrists choosing wisely? BJPsych advances 23:9–15. https://doi.org/10.1192/apt.bp.115.015271

3. England NHS (2020) Structured Medication Reviews and Medicines Optimisation. https://www.england.nhs.uk/publication/structured-medication-reviews-and-medici- nes-optimisation/ Zugegriffen: 13.01.2022

4. Jordan M, Marshall H (2010) Taking counselling and psychotherapy outside: Destruction or enrichment of the therapeutic frame? European Journal of Psychotherapy & Counselling 12:345–359. https://doi.org/10.1080/13642537.2010.530105

5. Lawrance E, Thompson R, Fontana G et al. (2021) The impact of climate change on men- tal health and emotional wellbeing: current evidence and implications for policy and practice. https://doi.org/10.25561/88568

6. Monsell A, Krzanowski J, Page L et al. (2021) What mental health professionals and organisations should do to address climate change. BJPsych Bull 45:215–221. https://doi.org/10.1192/bjb.2021.17

7. Cussans A, Harvey G, Kemple T et al. (2021) Interventions to Reduce the Environmental Impact of Medicines: A UK perspective. The Journal of Climate Change and Health 4:100079. https://doi.org/10.1016/j.joclim.2021.100079

8. Panu P (2020) Anxiety and the Ecological Crisis: An Analysis of Eco-Anxiety and Climate Anxiety. Sustainability 12:7836. https://doi.org/10.3390/su12197836

9. Hickman C, Marks E, Pihkala P et al. (2021) Climate anxiety in children and young people and their beliefs about government responses to climate change: a global survey. Lancet Planet Health 5:e863–e873. https://doi.org/10.1016/S2542-5196(21)00278-3

10. Nikendei C (2020) Klima, Psyche und Psychotherapie: Kognitionspsychologische, psychodynamische und psychotraumatologische Betrachtung einer globalen Krise. Psychotherapeut 65:3–13. https://doi.org/10.1007/s00278-019-00397-7

11. Guzmán CAF, Aguirre AA, Astle B et al. (2021) A framework to guide planetary health education. The Lancet Planetary Health 5:e253–255. https://doi.org/10.1016/S2542-5196(21)00110-8
